# Assessment of Macroscopic Properties Based on Microhardness Measurements Using the Example of the TiCoCrFeMn High-Entropy Alloy

**DOI:** 10.3390/ma19010118

**Published:** 2025-12-29

**Authors:** Dominika Przygucka, Krzysztof Karczewski, Zbigniew Bojar, Stanisław Jóźwiak

**Affiliations:** Faculty of Advanced Technologies and Chemistry, Military University of Technology, Sylwestra Kaliskiego 2, 00-908 Warsaw, Poland; krzysztof.karczewski@wat.edu.pl (K.K.); zbigniew.bojar@wat.edu.pl (Z.B.); stanislaw.jozwiak@wat.edu.pl (S.J.)

**Keywords:** high-entropy alloys, true hardness, young modulus

## Abstract

In this study, using the newly designed high-entropy alloy TiCoCrFeMn as an example, mathematical models for determining the true hardness from microhardness measurements in the regime dominated by elastic deformation were analyzed. It was found that the PSR model, which accounts for the variability in the elastic component of the applied indenter load, provides the best agreement with macroscopic hardness measurements. Analysis of the load variation law revealed a correlation between the Young’s modulus and the Meyer coefficient, which formed the basis for developing a model for determining this material parameter from microhardness measurements. The proposed methodology, applicable to small laboratory specimens, was shown to be consistent with the results obtained from crack-length measurements.

## 1. Introduction

In early-stage research, new materials are commonly investigated at a microscale due to limited sample availability, which enables only a preliminary evaluation of their structure, thermal stability, and selected mechanical properties. While structural characterization can be performed on very small specimens, mechanical testing is considerably more difficult. Consequently, hardness measurements—particularly the Vickers method—are widely used as a rapid, simple, and minimally destructive means of initial mechanical assessment. Although hardness is not a fundamental material constant, it correlates with tensile strength, yield strength, and elastic behavior [1]. As such, hardness provides insight into microstructural uniformity and the effectiveness of processing steps such as heat treatment [2], plastic deformation, or thermochemical processing. Hardness data are also applied to evaluate service-induced degradation and estimate remaining lifetime [3].

Empirical studies indicate that the hardness-to-tensile strength ratio typically ranges from 2.3 to 3.7. More specific relationships include [4]:

(1) Annealed materials: 3$\sigma_{YS\;}$< HV< 3$\sigma_{UTS}$;
(2) Work-hardened materials: HV≈ 3$\sigma_{YS}$;
(3) HPT-processed alloys: HV< 3$\sigma_{YS\;}$.

However, hardness results depend strongly on the measurement method, load, and indenter geometry. A reliable comparison is possible only under consistent testing conditions that satisfy Kick’s similarity law. Load-independent Vickers hardness requires similarity in plastic deformation, a condition rarely met in practice. Surface heterogeneity and indenter–material friction may further influence the results. These limitations are critical for very small samples, where macroscopic loads cannot be applied [1]. Microhardness testing at low loads increases the elastic contribution to indentation, thereby increasing measurement scatter. The well-known indentation size effect (ISE)—an apparent hardness increase with decreasing load—arises from the nonlinear partitioning of elastic and plastic deformation and from microstructure–stress field interactions. To minimize geometric errors, the specimen thickness should exceed 1.5 times the diameter (d), and the indentation spacing should be at least 3 times the diameter (d) [1,2].

**Figure 1:**
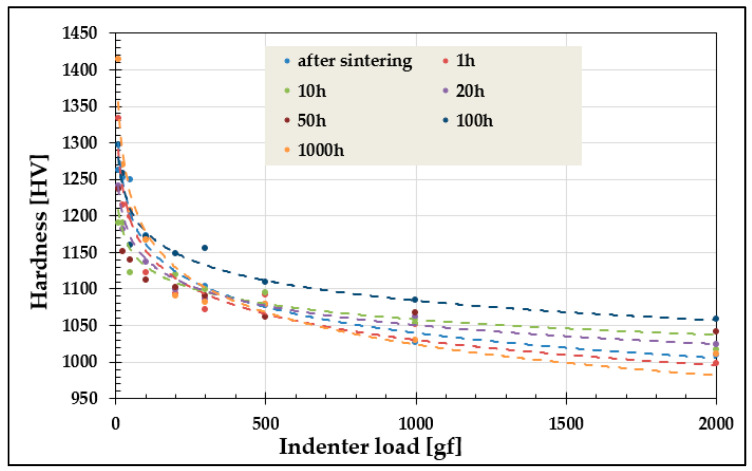
Change in hardness value as a function of Vickers indenter load for TiCoCrFeMn alloy after sintering and homogenizing annealing for up to 1000 h.

A pronounced ISE was observed in the TiCoCrFeMn high-entropy alloy examined in [5,6,7]. Sample dimensions precluded testing at loads above 5 kgf, necessary for compliance with Kick’s law. Consequently, microhardness was measured at 10–1000 gf, yielding hardness variations of up to 20% (~200 HV) across heat-treatment conditions (Figure 1). This strong ISE prevented the determination of macroscopic hardness, and consequently, hindered the estimation of strength for the alloy.

Despite their limitations, microhardness tests remain essential for assessing the basic mechanical properties of materials available only in very small quantities. This creates a need for methods that minimize the adverse influence of elastic deformation on hardness measurements, manifested as the indentation size effect (ISE). To address this issue, several empirical models have been developed and validated across numerous metallic materials, including Meyer’s law, the Hays–Kendall model, the Proportional Specimen Resistance (PSR) and Modified PSR (MPSR) models, and the Nix–Gao model [8,9,10,11,12,13,14,15,16,17,18,19]. However, comparative data evaluating the ability of these models to reproduce the true hardness across a wide spectrum of engineering materials are still limited.

In [6], difficulties in determining the hardness of the TiCoCrFeMn alloy were noted, and PSR-derived hardness values were subsequently used to establish the Hall–Petch relationship. Some models have also been applied to other high-entropy alloys, yielding more reliable results that are independent of sample geometry or scale effects. For example, the Nix–Gao model was used to interpret hardness data for the Cantor alloy CoCrFeMnNi, showing good agreement with the ISE for indentation depths above 1 μm. At lower depths, deviations were observed due to additional deformation mechanisms, such as geometrically necessary dislocations (GNDs) [20].

Nevertheless, high-entropy alloys constitute a relatively new class of materials, and there remains a lack of studies focused on determining true hardness from microscale samples, for which conventional macroscopic hardness testing is impossible. Such analyses are crucial not only for estimating tensile strength and yield strength but also for deriving other fundamental mechanical properties from microhardness measurements [21], including:

- Work-hardening exponent *n* (from Meyer’s law);
- Qualitative stress–strain behavior;
- Indentation creep;
- Coating adhesion;
- Stress intensity factor for brittle materials via crack analysis;
- Young’s modulus.

These considerations motivated the present work, which aimed to evaluate the accuracy of established hardness-correction models for determining true hardness from microhardness data. Furthermore, while hardness depends on numerous material and testing factors, Young’s modulus—being a fundamental elastic constant—is independent of material condition. Determining this parameter is similarly challenging when only very small quantities of material are available, as conventional methods (tensile testing, ultrasonic measurements) require large specimens, and nanoindentation techniques are not always accessible. Given the correlation between elastic and plastic properties described by the stress–strain curve σ = f(ε), and considering the influence of elastic deformation on hardness variation in the ISE regime, this work also proposes a methodology for estimating Young’s modulus from Vickers microhardness measurements. The approach is based on Meyer’s law and Kick’s similarity principle, enabling the determination of this fundamental elastic constant using only microscale specimens.

## 2. Materials and Methods

### 2.1. Material

The material investigated in this study was a newly developed five-component high-entropy alloy (HEA) based on titanium, with the nominal composition Ti24Co19Cr19Fe19Mn19. Due to its dual-phase microstructure, comprising a BCC solid solution and an HCP phase, the alloy offers potential for applications in nuclear energy systems [22,23] and high-temperature superconductors [24,25]. The base composition was produced using conventional powder metallurgy due to the possibility of precise control over the chemical composition. Initial powders of Ti, Co, Cr, Fe, and Mn with an average particle size of 45 μm were milled for 5 h under argon in a Fritsch Pulverisette 7 Premium planetary ball mill (Fritsch International, Idar-Oberstein, Germany). Steel bearing balls made of 100Cr6 with a diameter of 10 mm, at an 8:1 ball-to-powder ratio, were used as the grinding media. The resulting powder mixtures were consolidated using modified spark plasma sintering (SPS), specifically the Upgraded Field Assisted Sintering Technology (U-FAST) (Genicore, Warsaw, Poland). The compacted material was subjected to short, microsecond pulses of high current (~2000 A) and a pressure force of 13 kN, using a heating rate of 50 °C/min to densify the powder mixture. The sintering process was carried out at 1050 °C for one minute. Afterwards, the sinters with a diameter of 20 mm and a height of 10 mm were annealed in a protective argon atmosphere at 1000 °C for 1, 10, 20, 50, 100, and 1000 h to evaluate the structural stability of the resulting phase and grain structure. Metallographic samples for assessing grain size were ground on SiC abrasive papers with grit sizes ranging from 600 to 4000, and then polished using cloths and 3 μm and 0.25 μm diamond suspensions, followed by a 0.1 μm silica suspension.

**Figure 2:**
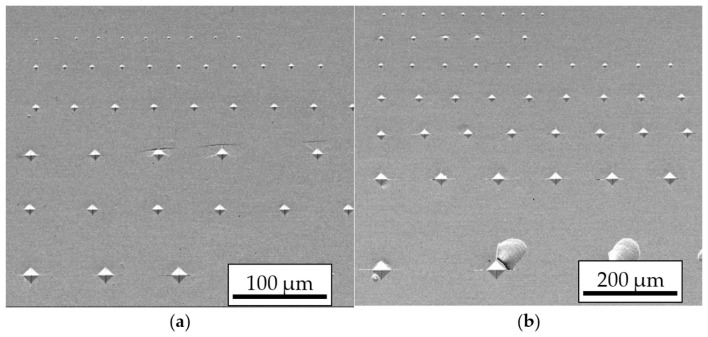
Example image of the indentation distribution map obtained by the Vickers method for loads in the range of 10–300 gf (**a**) and for loads of 50–2000 gf (**b**).

To evaluate hardness—the key mechanical parameter addressed in this work—Vickers microhardness measurements were performed on metallographic cross-sections using a Shimadzu HMV-G hardness tester (Shimadzu Corporation, Kyoto, Japan). Due to the small sample size, microhardness indentations were made under loads of 10, 25, 50, 100, 200, 300, 500, and 1000 gf, each applied for 10 s. Representative indentation maps are shown in Figure 2a,b.

### 2.2. Research Problem and Methodology

It is well established that for applied loads below 1 kgf, the measured hardness is strongly load-dependent due to the increasing contribution of elastic deformation. In the present study, a pronounced sensitivity of hardness to the applied load was observed, and a variable-hardness region was identified for loads between 10 and 1000 gf (Figure 1), corresponding to the well-known indentation size effect (ISE) [9,18,26,27,28,29,30,31].

**Figure 3:**
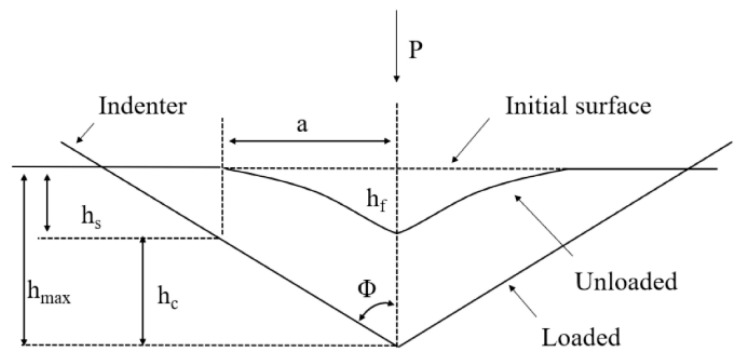
Change in the size of the indentation during hardness measurements in the ISE area [15,32].

The mechanism of this phenomenon is illustrated in Figure 3. When the indenter is loaded with force F, it penetrates the material to a depth h, representing the combined elastic and plastic deformation. Upon unloading, the material elastically recovers, reducing the indentation depth by h_c_ and resulting in a final residual depth h_f_ and an associated diagonal length 2a [15,32]. For materials of constant stiffness—characterized by Young’s modulus—the relative contribution of elastic recovery increases as the applied load decreases, leading to an apparent increase in hardness.

The observed divergence in hardness values for the same material under different loads raises a fundamental question: How can the true hardness be determined from measurements obtained within the ISE regime?

### 2.3. Determination of Hardness Closest to the True Value

**Figure 4:**
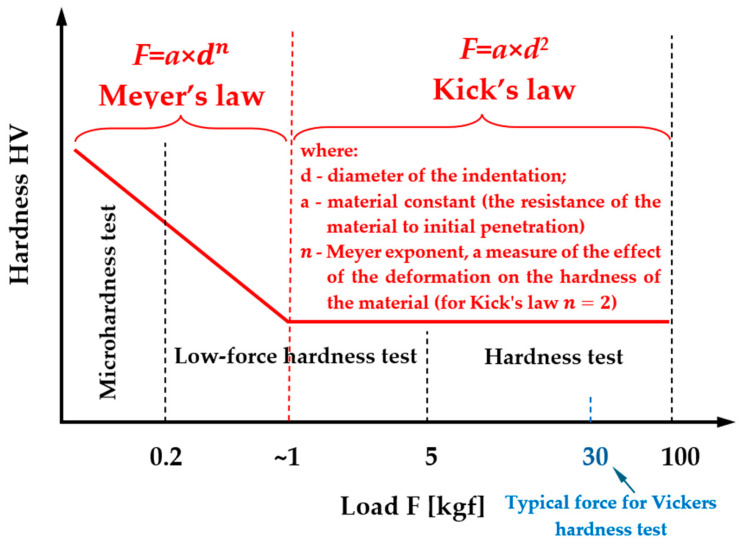
Areas of occurrence of Meyer and Kick’s law against the background of the division of hardness types depending on the indenter load in accordance with International Standard ISO 6507-1:2018(E) [33].

The determination of hardness closest to the true material value is described by Kick’s similarity law, which applies primarily to macroscopic hardness measurements (Figure 4). This law states that the dimensions of the indentation are proportional to the applied load, assuming that the indentation results solely from plastic deformation, with the elastic component neglected. Kick’s law is therefore valid under high loads, in contrast to Meyer’s law, which describes the load-dependent behavior in the microhardness regime (F < 1 kgf) where elastic deformation contributes significantly.

Meyer’s law additionally defines the Meyer exponent *n*, which characterizes the degree of strain hardening. In the Meyer framework, *n* < 2 is typical, indicating that microhardness increases as the applied load decreases. Kick’s law is satisfied for *n* = 2, corresponding to load-independent hardness [26].

The influence of elastic deformation and the resulting indentation size effect (ISE) can be mitigated using various analytical relationships and models proposed in the literature, although their accuracy differs significantly. In this work, the effectiveness of several commonly referenced models in reducing the elastic contribution was compared. The discussion naturally begins with Meyer’s law, which is the most frequently applied framework in studies addressing load-dependent hardness.

**Figure 5:**
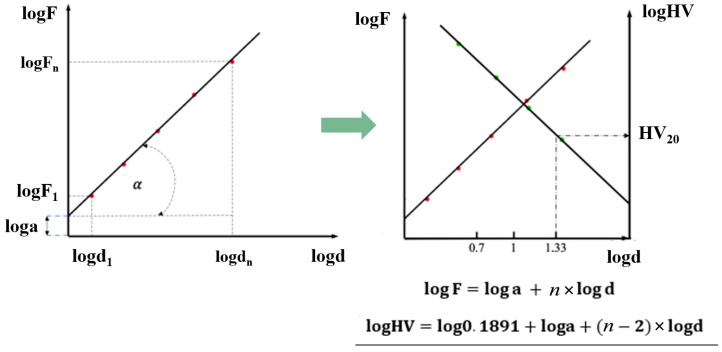
Determination of hardness values according to Meyer’s law for a constant indentation diagonal based on microhardness measurement results [34,35].

Meyer proposed that hardness should be determined for selected fixed indentation diagonals—typically 5, 10, or 20 µm—depending on the measured indentation range, thereby ensuring a constant level of plastic deformation (Figure 5).

However, the application of Meyer’s method still results in hardness values within the ISE region, as will be demonstrated in this study. This approach is therefore suitable only for comparing the hardness of different phases within a material (e.g., a ductile solid solution versus an intermetallic phase) or for comparing multiphase materials with differing Young’s moduli.

Other methods that allow for the determination of true material hardness, minimizing the influence of elastic deformation, include the previously mentioned models: Hays–Kendall, PSR, MPSR, and Nix–Gao. Their characteristics and the procedures for determining hardness according to each model are summarized in Table 1.

#### 2.3.1. Hays–Kendall Model

The authors of the model assumed that the total force consists of the force required to overcome elastic deformation, together with the plastic force, and that there exists a minimum applied load, W, necessary to initiate plastic deformation—below this threshold, only elastic deformation occurs [16,19]. Undoubtedly, the Hays–Kendall model enables the estimation of a material’s true hardness based on microhardness measurements. However, assuming a constant elastic force within the nonlinear elastoplastic deformation range, while the indentation area varies with the applied load, appears to be an overly simplistic approach and may introduce significant errors [19,31,36,37].

#### 2.3.2. Proportional Specimen Resistance (PSR) Model

The PSR model, proposed by Li and Bradt, can be regarded as a modified and alternative approach to the Hays–Kendall method for eliminating the influence of elastic deformation in the ISE region when determining true hardness [18,31].

Based on indentation load–displacement curves recorded for various materials, Li and Bradt suggested that the elastic resistance of the material, *W* is not constant, as assumed by Hays and Kendall [18], but increases proportionally with the indentation size. Furthermore, the material is modeled as a spring whose resistance to the indenter grows with the indentation size, accounting for both internal material friction and friction between the indenter and the specimen [19,38,39]. The PSR approach enables a more accurate determination of the true hardness by correcting for load-dependent elastic effects, particularly in microhardness measurements where the ISE is significant.

#### 2.3.3. Modified Proportional Specimen Resistance (MPSR) Model

The MPSR model builds on the PSR approach by considering that the total load applied to the indenter consists of several components: the force required to overcome the resistance of the material’s surface layer, the strengthening effect in the near-surface region related to residual stresses induced during surface preparation, the elastic component, and the component responsible for permanent plastic deformation [40,41]. To account for residual surface stresses arising from grinding and polishing during sample preparation, the MPSR model introduces an additional term, “*a*_0_”, representing a constant associated with these stresses. The elastic–plastic interaction of abrasive grains on the metallographic surface is considered analogous to the action of a series of single-point indenters located closely together [18]. These factors can significantly influence microhardness measurements. Therefore, the MPSR model incorporates their effect to provide a more accurate estimation of the true material hardness based on microhardness data [42].

#### 2.3.4. Nix & Gao Model

According to Nix and Gao, the origin of the Indentation Size Effect (ISE) is not unequivocally defined; however, a widely accepted explanation—supported by the correlation between ISE and the hardness-to-elastic modulus ratio—is its relation to strain gradients and the density of dislocations generated during indentation. Within the deformed zone, dislocations characterized by specific Burgers vectors are created [28,43]. In the dislocation-based model, geometrically necessary dislocations (GNDs) increase with decreasing indentation depth, leading to higher local strengthening and, consequently, increased hardness [26]. By applying Taylor’s model, which assumes uniform strain distribution throughout the sample [44], Nix and Gao proposed a relationship between indentation hardness *H* and indentation depth ℎ [25,26,45]. The empirical relation, along with the graphical procedure for determining the true hardness *HV*_*T*_ using all models, is presented in Table 1.

While many studies employ the above models, there is limited information regarding their accuracy in reproducing the true hardness values. Therefore, in the present study, an analysis was planned to evaluate the precision of estimating the true hardness from microhardness measurements in the ISE regime, corresponding to the Kick’s similarity law. This approach enables the verification and selection of the most suitable model for further correlation between structural and mechanical properties of the investigated materials, providing practical guidance for optimizing experimental methodologies in material characterization.

**Table 1:** Summary of relationships used when estimating the actual hardness for selected empirical models based on [46].

Model	Relation	Regression Equation: $\;Y=a_{0}+a_{1}\times X$		${HV}_{T}$
		Y	X	
Hays-Kendall	$F=a_{0}+a_{1}\times d^{2}$ 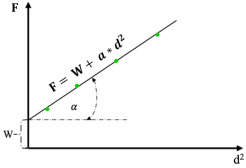	F	$d^{2}$	$1.8544\times a_{1}$
Li-Bradt (PSR)	$F=a_{0}\times d+a_{1}\times d^{2}$ $\;\frac{F}{d}=a+a_{1}\times d$ 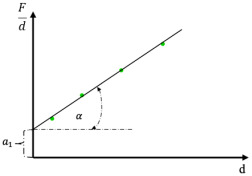	$\frac{F}{d}$	d	$1.8544\times a_{1}$
MPSR	$F=a_{0}+a_{1}\times d+a_{2}\times d^{2}$ 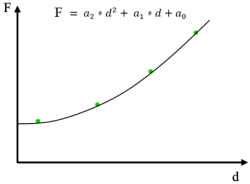	Regression equation $\;Y=a_{0}+a_{1}\times X+a_{2}\times X^{2}$		$1.8544\times a_{2}$
		Y	X	
		*F*	*d*	
Nix-Gao	$H=H_{0}\times \sqrt{1+\frac{d^{*}}{d}}$ $\;H^{2}=H_{0}^{2}+H_{0}^{2}\times \frac{d^{*}}{d}$ 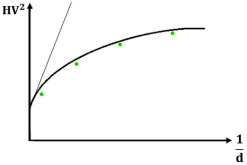	$H^{2}$	$d^{-1}$	$\sqrt{a_{0}}$

## 3. Results

As reported in [5,6,7], the high-entropy alloy TiCoCrFeMn, produced via powder sintering, exhibits a dual-phase microstructure consisting of a BCC solid solution and a C14 Laves phase. Due to the inherently slow diffusion in this system, achieving a chemically and phase-stable microstructure requires prolonged homogenization. Consequently, to accurately evaluate the effect of thermal treatment duration on the structural and mechanical properties, it is essential to determine hardness as a quantitative parameter. This enables the assessment of macroscopic property changes induced by technological processing, including extended thermal treatment.

**Figure 6:**
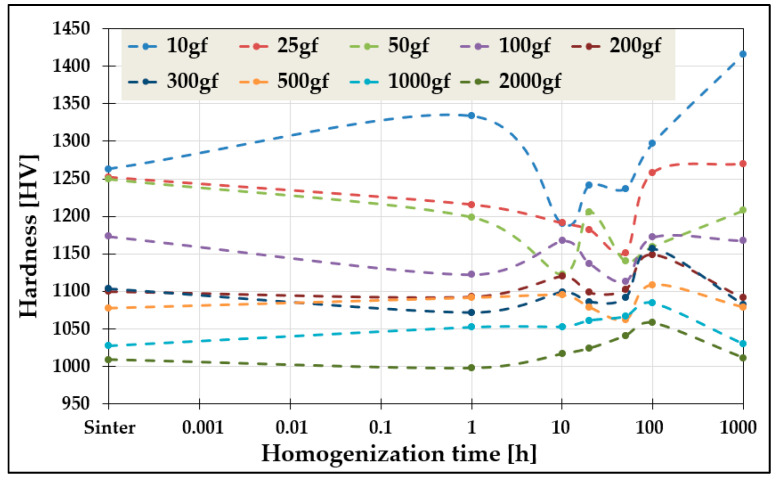
Differences in the change in hardness value of the high-entropy TiCoCrFeMn alloy as a function of homogenizing annealing time for different values of the Vickers indenter load.

Nevertheless, the microhardness measurements of the studied material (Figure 6) reveal significant differences—both qualitative and quantitative—in the evolution of hardness depending on the applied load. As mentioned, the designed alloy exhibits a dual-phase structure, consisting of a solid solution and a hard Laves phase, which increases the hardness to an unusually high level for high-entropy alloys, exceeding 1000 HV [5,6,7].

**Figure 7:**
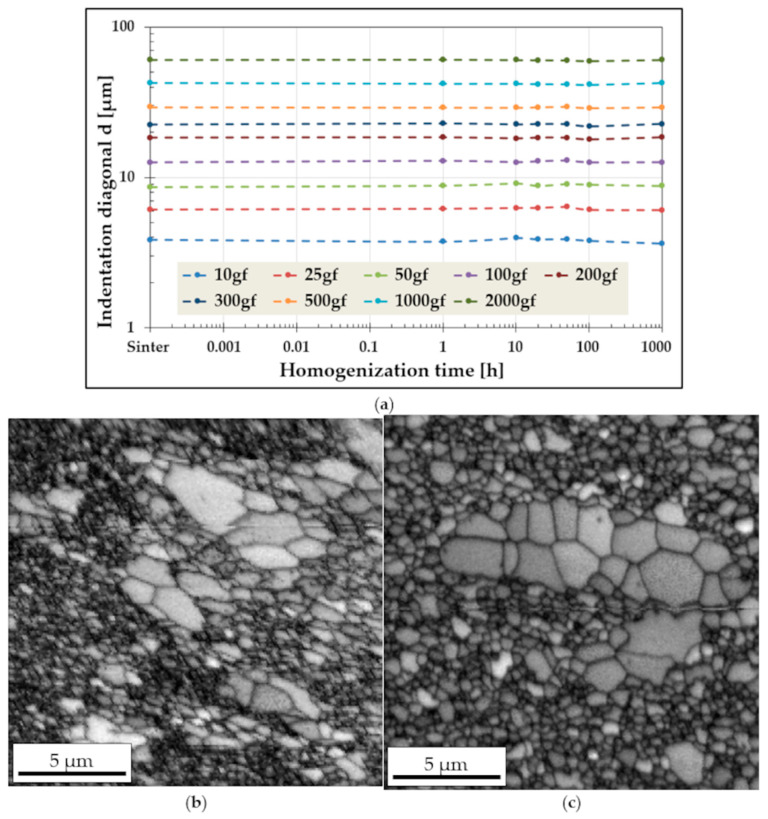
Changes in the size of the indentation diagonals as a function of homogenization time for different load values (**a**) with a view of the grain structure of the tested high-entropy TiCoCrFeMn alloy after the sintering process (**b**) and homogenization at 1000 °C for 1000 h (**c**).

During the annealing process, changes in the chemical composition within the main phase constituents lead to fluctuations in hardness values, which are more pronounced at lower indenter loads. This behavior is primarily due to the relative size of the indentation compared to the microstructural grain size. This effect is clearly illustrated in Figure 7, which shows the evolution of indentation diagonals as a function of homogenization time for different loads (Figure 7a), alongside the microstructure of the alloy (Figure 7b,c).

At low loads, for example 10 gf, the indentation diagonal measures approximately 4 μm, meaning that the measurement statistically samples only a small number of grains. Consequently, the hardness measurement is highly localized and may reflect regions with significantly different phase composition, morphology, or properties. As the load increases, the indentation size also increases—for instance, at 1000 gf, the diagonal reaches approximately 40 μm—effectively averaging the measurement over a much larger area, i.e., a greater number of microstructural elements. This averaging effect results in substantially reduced hardness fluctuations at higher loads, as observed for 1000 and 2000 gf in Figure 6. The influence of grain size on hardness and its consistency with the Hall–Petch relationship were discussed in detail in [6].

Nevertheless, the above analyses of the microhardness measurements, characterized by a significant scatter ranging from approximately 1000 HV up to 1400 HV due to the Indentation Size Effect (ISE), necessitate approaches that enable the determination of a hardness value as close as possible to the true material hardness, in accordance with Kick’s law of similarity—i.e., under loads where the influence of elastic deformation is negligible. Therefore, the hardness value measured at a load of 30 kg was assumed to represent the true hardness. This can be achieved using the methodology proposed by Meyer, which involves comparing hardness values calculated for a constant indentation size, as well as the empirical models summarized in Table 1.

**Figure 8:**
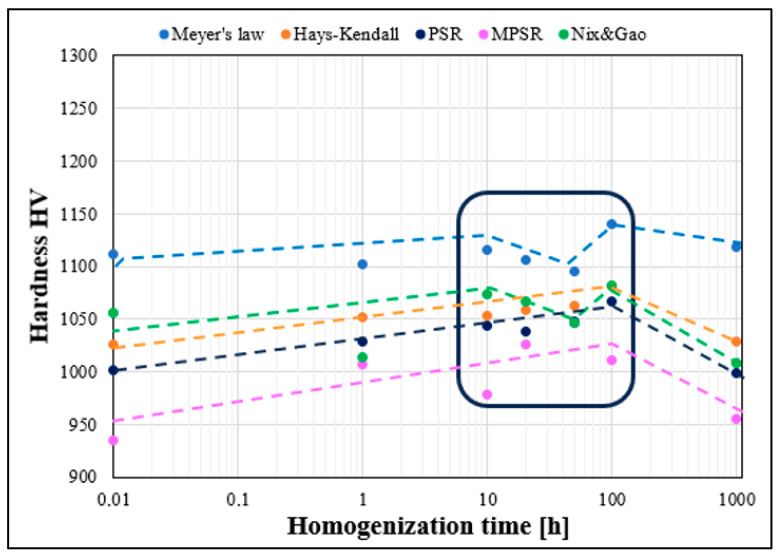
Changes in the actual hardness of the TiCoCrFeMn alloy as a function of homogenization time determined for the analyzed empirical models.

Accordingly, the obtained microhardness results were recalculated using Meyer’s methodology and the empirical models of Hays & Kendall, PSR, MPSR, and Nix & Gao. The results are compiled in Table 2 and illustrated in Figure 8. It can be observed (Figure 8) that the true hardness values determined using Meyer’s methodology reach the highest values while simultaneously deviating from the hardness levels predicted by the other empirical models. This is a consequence of the approach of comparing hardness at a fixed indentation diagonal, which in the present analysis was set at 20 μm, corresponding to a constant plastic strain.

However, for the alloy under investigation, this diagonal size occurs under loads of 200–300 gf, which, according to the data presented in Figure 1, places these results within the ISE region—i.e., in the range influenced by elastic deformation. Therefore, the Meyer methodology can only be reliably used for comparing the hardness of two distinct phases or materials determined in this manner, while keeping in mind that, particularly for high-hardness materials, this model may not fully eliminate the effects of ISE. Consequently, its use for determining the true hardness value is highly problematic and limited.

For the other empirical models, namely Hays & Kendall, PSR, MPSR, and Nix & Gao, the obtained hardness values, similarly to the microhardness measurements (Figure 6), exhibit fluctuations within the 10 to 100 h homogenization period of the investigated alloy. This observation demonstrates that the proposed models are qualitatively sensitive to structural and mechanical changes occurring in the material. Nevertheless, a significant scatter of the results, reaching up to 10%, is still observed, depending on the model applied.

Therefore, to determine which of the analyzed microhardness-based hardness estimation models most accurately reflects the true hardness, the methodology was repeated using materials with known HV30 hardness values. For this purpose, certified steel standards with known hardness values—759 HV30 ± 16, 51.3 HRC, 228 HV0.1 ± 9—and a cutting insert made of sintered carbide with a hardness of 1472 HV30 were used. The selected hardness range for the reference materials, 200–1500 HV, was intended to cover a broad spectrum of hardness values for commonly used engineering materials, to identify which model is the most universal.

Since the available certified hardness standards were provided for different hardness measurement methods (Vickers and Rockwell), the Vickers hardness value was determined for all reference samples using a 30 kgf load, ensuring values within the macroscopic hardness range, independent of the applied load, as described by Kick’s law of similarity (Table 3). The obtained hardness values of the selected standards were then adopted as reference values for comparison with the results obtained from the analysis of the empirical models.

Microhardness measurements were performed on the selected reference standards under loads of 10, 25, 50, 100, 200, 300, 500, and 1000 gf. For each load level, ten indentations were made, and the average diagonal lengths obtained for each load are presented in Table 4.

**Figure 9:**
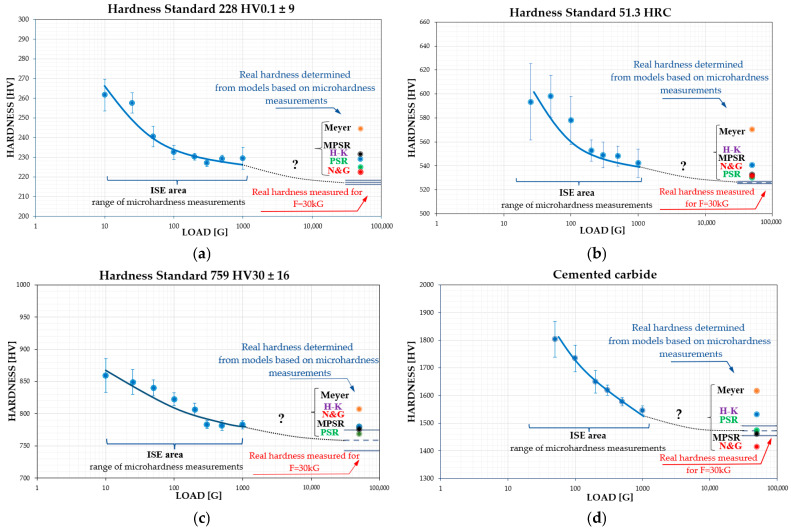
Evolution of hardness changes from the ISE range valid for Meyer’s law to the actual hardness valid for Kick’s law on a macroscopic scale, which was determined using the theoretical models: Hays and Kendall, PSR, MPSR, and Nix-Gao for steel standards: 228 HV0.1 ± 9 (**a**), 51.3 HRC (**b**), 759 HV30 ± 16 (**c**) and for a carbide cutting insert with hardness of 1472 HV30 (**d**).

By analyzing the obtained results according to the methodology applied to the studied high-entropy alloy, the actual hardness values of the reference standards were determined based on microhardness measurements in the ISE region and compared with the true hardness measured under a 30 kgf load (Table 5 and Figure 9).

The comparison of hardness values determined from empirical models within the range governed by Kick’s law with the hardness measured under a 30 kgf load allowed for the calculation of measurement errors for each model and each reference standard. This, in turn, enabled the determination of the average error for each model across the analyzed hardness range of 216–1454 HV (Table 6). The analysis conducted for the individual models clearly demonstrated that the PSR model exhibited the lowest average relative error at 1.54%, while the Hays–Kendall model showed the highest at 3.40%.

**Figure 10:**
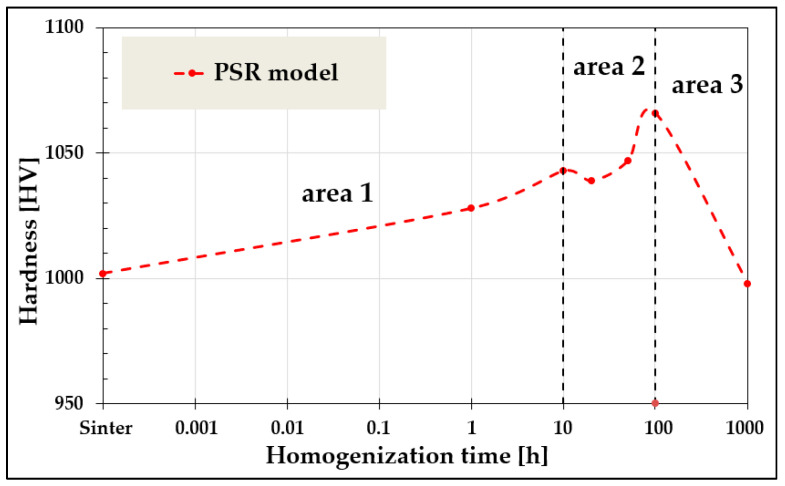
Changes in the actual hardness values of the high-entropy TiCoCrFeMn alloy as a function of homogenization time determined using the PSR model based on microhardness measurements.

The analysis of actual hardness changes corresponding to HV30, obtained using the PSR model (Figure 10), correlates closely with the changes in chemical composition and phase structure of the studied alloy as a function of homogenization time, as described in detail in [5,6]. The initial slow increase in hardness observed for homogenization times up to 10 h (Area 1) is attributed to the restructuring of solid solutions formed primarily from Cr into the multicomponent BCC high-entropy solid solution. In the subsequent time interval, 10–100 h (Area 2), pronounced fluctuations in chemical composition associated with the formation of the final two-phase matrix also result in non-monotonic hardness variations. Further continuation of the heat treatment process (Area 3) promotes grain growth of the formed phases, leading to a gradual decrease in hardness.

### Methodology for Determining Young’s Modulus from Microhardness Measurements

**Figure 11:**
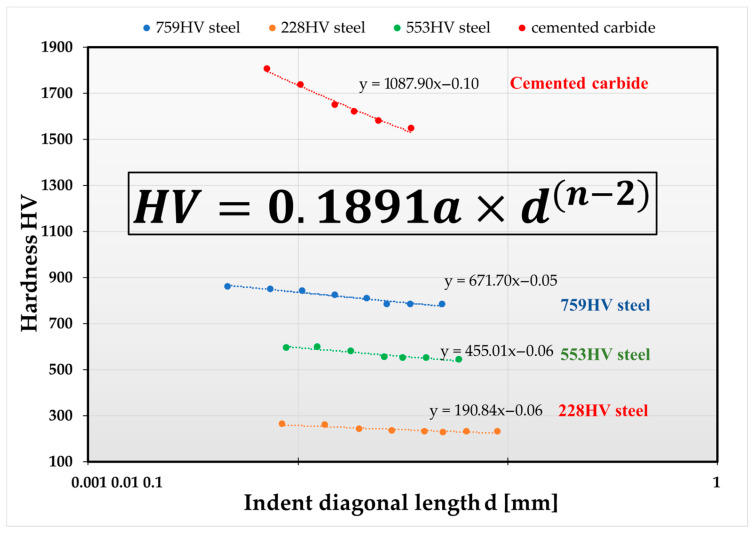
The slope of the hardness curves determined from the ISE area for the standard samples according to the law of variable hardness.

Analyzing the fit of models used to determine the actual hardness from microhardness measurements performed in the ISE region, it was observed that different material groups exhibit varying slopes of the lines obtained from Meyer’s law of variable hardness. Furthermore, it was noted that samples made from the same engineering alloy, such as steel, despite significant differences in hardness values, have identical slopes determined by Meyer’s exponent “*n*” (Figure 11).

It is well known that a constant, invariant, and material-independent mechanical property is the Young’s modulus, which serves as a measure of the stiffness of an engineering material. For steel, this critically important parameter ranges from E = 200 to 210 GPa. This observation formed the basis for the development of an original model enabling the estimation of Young’s modulus from microhardness measurements performed within the ISE region.

Knowledge of the Young’s modulus is of paramount importance in engineering practice, both in the field of investigating the mechanical properties of structural materials and in the design of new composite materials. Designing the mechanical properties of such composites is based on knowing the Young’s modulus of the individual structural components, which allows for determining, using mixture rules or more sophisticated models such as the Halpin–Tsai model, the overall stiffness described by the Young’s modulus of the newly developed structural material.

Of course, there are well-established methods for determining this parameter, such as static mechanical testing or ultrasonic measurements; however, these methods require large, bulky specimens. As mentioned in the introduction, at the stage of designing new materials, techniques such as additive manufacturing or powder metallurgy are often used to produce laboratory-scale samples for microstructural and spectroscopic characterization. In such cases, performing conventional mechanical tests, including the determination of Young’s modulus, is practically impossible.

There are, however, nanoindentation methods that allow for estimation of this material parameter using micro- or even nano-scale loads. Applying a force to the surface of micro-samples produces both elastic and plastic deformation. Upon unloading, the elastic deformation disappears, leaving only the plastic deformation. The slope of the unloading curve is then used to calculate Young’s modulus, provided the modulus of the indenter is known. Despite the apparent simplicity of this procedure, its application is strongly limited by the cost of the required apparatus and the specialized software needed to analyze the results.

There is also a known model for determining Young’s modulus based on microhardness measurements performed using the Knoop method. According to the data presented in [38], the influence of elastic deformations on changes in the Knoop indentation diagonals can be expressed as follows:

(1) $$\frac{W}{L}=\frac{W^{\prime }}{L^{\prime }}-0.45\times \frac{HK}{E}$$

where:

$\frac{W}{L}$—ratio of the shorter diagonal to the longer diagonal of the indentation, measured after unloading;
$\frac{W^{\prime }}{L^{\prime }}$—ratio of the shorter diagonal to the longer diagonal of the indenter, equal to 0.1406;
*HK*—Knoop hardness;
*E*—Young’s modulus.

Equation (1) appears to allow for a straightforward calculation of Young’s modulus from Knoop hardness measurements. However, as noted by the authors in [17], the resulting value is subject to significant error. For example, the Young’s modulus calculated using Equation (1) for β-SiAlON ceramics is 160 GPa, whereas the actual value, depending on density, should lie within the 200–300 GPa range. This discrepancy motivated an evaluation of the validity of Equation (1) using a material with a known Young’s modulus. To this end, Knoop microhardness measurements were performed on a steel reference sample with a hardness of 759 HV30, which had been previously used to verify the accuracy of models determining true hardness from Vickers microhardness measurements.

**Figure 12:**
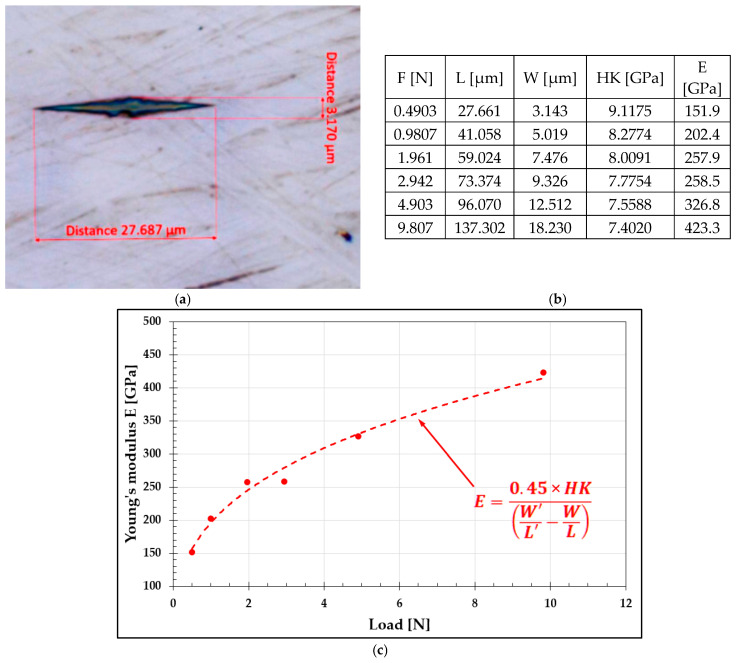
Example view of the Knoop indentation (**a**) along with the results of microhardness measurements of the steel standard sample (**b**) and changes in the Young’s modulus E value as a function of the applied indenter load (**c**).

The analysis of the obtained results (Figure 12) clearly demonstrated that the Young’s modulus calculated using Equation (1) strongly depends on the applied indenter load. It is well known that for steel, this parameter remains practically constant, with E = 200–210 GPa, regardless of the heat treatment state. In the analyzed case, however, the calculated values ranged from 150 to 400 GPa, which effectively disqualifies the use of this equation for determining a unique and accurate value of Young’s modulus.

**Figure 13:**
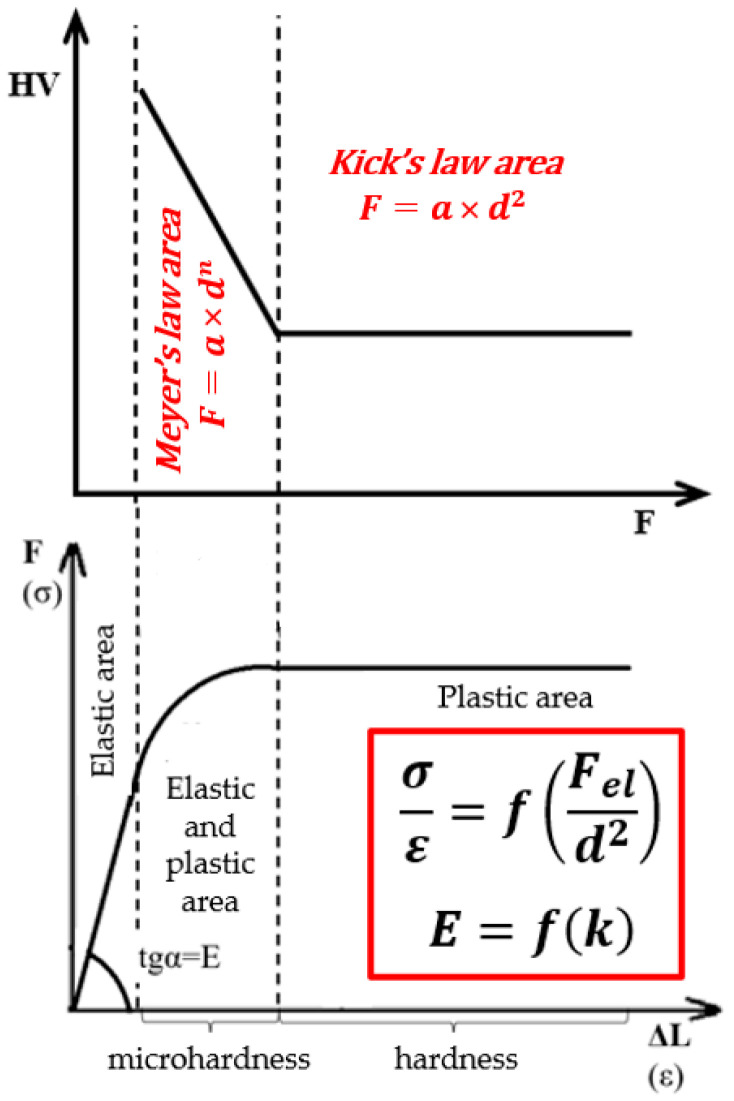
Comparison of the change in hardness measured by the Vickers method as a function of the applied load HV = f(F) with the classical stress–strain curve σ = f(ε).

In the proposed methodology, changes in Vickers hardness as a function of applied load, HV = f(F), were compared with the classical stress–strain curve, σ = f(ε) (Figure 13).

According to Meyer’s theory, in the elastic–plastic region (ISE), the applied force can be determined from the following relationship:

*F* = *a* × *d^n^* (2)

whereas in the region dominated by plastic deformation, the force is determined by Kick’s law, expressed by the following relationship (3):

*F* = *a* × *d*^2^ (3)

The elastic force can therefore be expressed as the difference between the elastoplastic component and the plastic component:

(4) $$F_{el}=\left(F_{el}+F_{pl}\right)-F_{pl}$$

Substituting Meyer’s relation (2) and Kick’s law (3) into the expression above yields a formula that allows for calculation of the elastic forces in the ISE region, dependent on the indentation diagonal (5):

(5) $$F_{el}={ad}^{n}-{ad}^{2}$$

After algebraic manipulations, this leads to the final form (6):

(6) $$F_{el}=a\left(d^{n}-d^{2}\right)\;\;F_{el}=ad^{2}(d^{n-2}-1)$$

By dividing Equation (6) by the square of the indentation diagonal, we obtain the relationship (7):

(7) $$\frac{F_{el}}{d^{2}}=a\left(d^{n-2}-1\right)\;\;\frac{F_{el}}{d^{2}}=a\times d^{n-2}-a$$

By comparing Equation (7) with the formula for Meyer hardness (Figure 5 and Figure 12), the ratio of the elastic component of the indenter load to the square of the indentation diagonal can be expressed as a relationship (8):

(8) $$\frac{F_{el}}{d^{2}}=\frac{HV}{0.1891}-a$$

Since the *HV* hardness of a given material is a constant, and the force causing a unit indentation, “*a*,” is also constant for that material (Figure 5), the right-hand side of the expression can be replaced by a constant “*k*,” referred to as the correlation coefficient (9):

(9) $$k=\frac{F_{el}}{d^{2}}$$

The material properties in the elastic region are characterized by the Young’s modulus, which also depends on the magnitude of the elastic force and the elastic strain (10).

(10) $$E=\frac{\sigma }{\varepsilon }=\frac{F_{el}\times L_{0}}{S\times \Delta L}$$

Hence, it seems reasonable to assume that for a given material, the value of the Young’s modulus should be functionally related to the correlation coefficient “*k*” (11):

*E* = *f*(*k*) (11)

**Figure 14:**
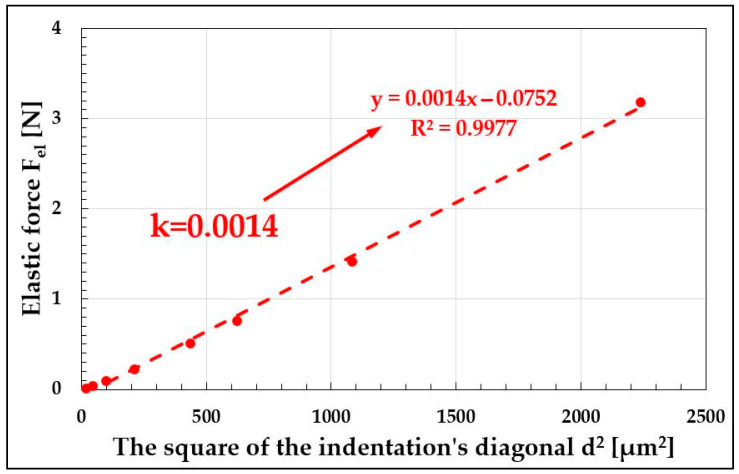
Method of determining the correlation coefficient “k” on the example of the 759HV steel standard.

To verify the validity of this assumption, microhardness measurements were conducted on a series of typical structural alloys with known Young’s modulus values, ranging from metals such as lead, aluminum, copper, molybdenum, and tungsten, to steels, titanium, copper, and aluminum alloys. For each material, the indentation diagonal was measured under a given load, and following the methodology presented in Figure 5, the Meyer exponent “*n*” and the unit load “*a*” were determined. According to Equation (5), this allowed for the calculation of the elastic component for the applied indenter load. Each elastic component was then divided by the indentation area “*d*^2^”, enabling the construction of the *F_el_* = *f*(*d*^2^) plot, from which the correlation coefficient “*k*” was determined via linear regression (Table 7 and Figure 14).

**Figure 15:**
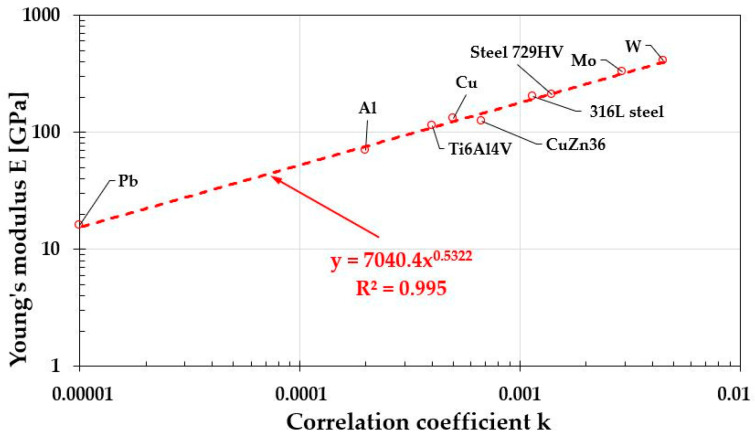
Characteristics of the dependence of the Young’s modulus E value on the correlation coefficient “k” determined from hardness measurements.

**Figure 16:**
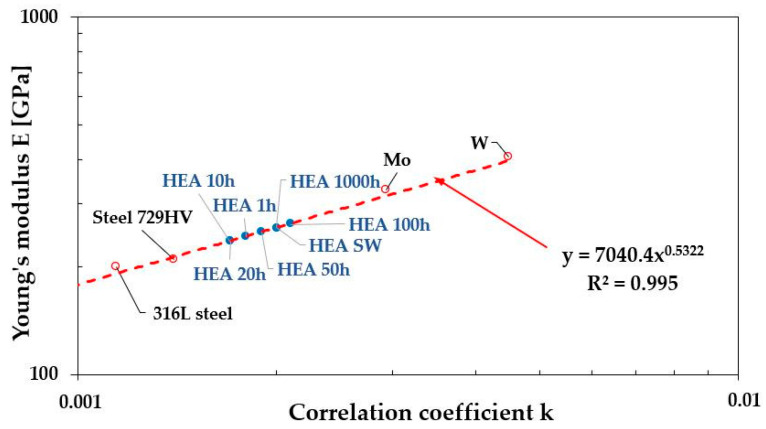
Calculation of the Young’s modulus E as a function of homogenization time of the tested high-entropy alloy from the determined characteristic E = f(k).

The correlation coefficient “k” values determined for metals and their alloys, whose Young’s modulus ranged from 16 GPa for lead to 405 GPa for tungsten (Table 8), enabled the construction of a characteristic relationship between the Young’s modulus E of a given material and the correlation coefficient “k” obtained from hardness measurements (Figure 15). Determining the function E = f(k) subsequently allowed for calculation of the Young’s modulus of the high-entropy alloy after various durations of homogenization annealing (Table 8 and Figure 16).

**Figure 17:**
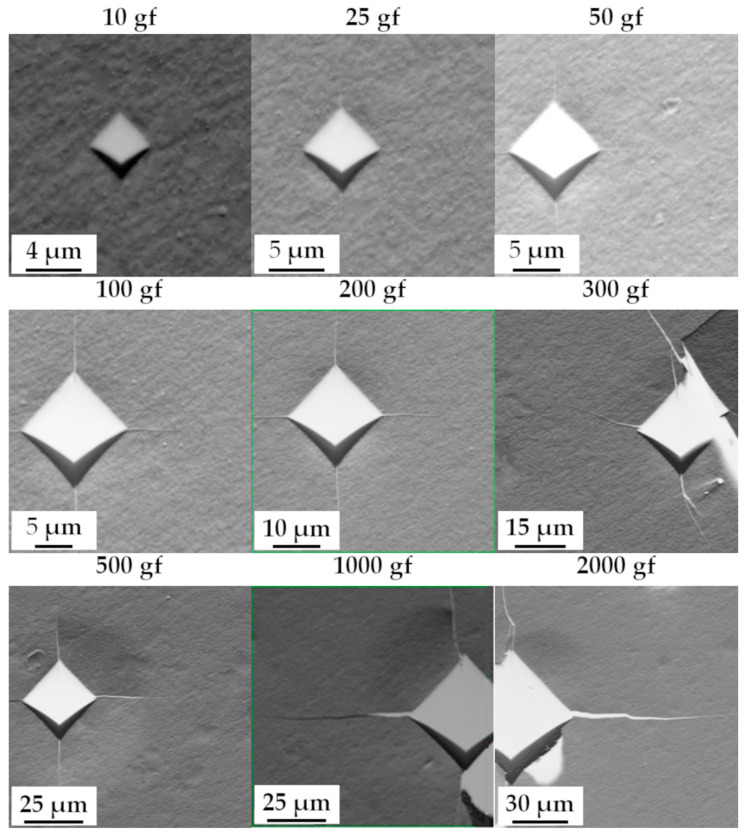
Example images of cracks and plastic deformation fields around the indentations for the TiCoCrFeMn alloy after the sintering process for the loads used during microhardness measurements using the Vickers method.

To verify the validity of the developed model, the Young’s modulus values of the high-entropy alloy TiCoCrFeMn obtained using the proposed method were compared with the results derived from measurements of the stress intensity factor *K_I_*. This parameter was determined from the lengths of cracks formed during hardness testing (Figure 17) using a modified Shetty model, adapted explicitly for brittle materials and Palmqvist cracks (Equation (12)) [47]. The obtained stress intensity factor values were then substituted into the Nihara Equation (13) [48], which allowed for an indirect determination of the Young’s modulus for the alloy in its various homogenization states (Table 9).

(12) $$K_{I}=0.0937\times {\left[H_{0}\frac{\left(P-P_{0}\right)}{4l}\right]}^{0.5}$$

(13) $$K_{I}=0.0089\times {\left(\frac{E}{H_{0}}\right)}^{0.4}\times \frac{P}{a\times l^{0.5}}$$

where:

$K_{I}$—fracture toughness [MPa × $m^{0.5}$];
$H_{0}$—Vickers hardness [MPa];
*P*—load applied during the Vickers hardness test [N];
*P*_0_—critical load at which a crack forms [N];
*l*—average crack length [μm];
*a*—half of the average diagonal length of the indentation [μm].

**Table 9:** Example values of crack length measurements for the TiCoCrFMn alloy homogenized at 1000 H, used to determine the stress intensity factor KI from the Shetty et al. model, and then the Young’s modulus E from the Nihara et al. model.

				K_I_ Determined Using the Modified Shetty et al. Model	Young’s Modulus E Determined from the Nihara et al. Model $K_{I}=0.0089\times {\left(\frac{E}{H_{0}}\right)}^{0.4}\times \frac{P}{a\times l^{0.5}}$
P [N]	a [μm]	l [μm]	H0 [MPa]	$K_{I}=0.0937\times {\left[H_{0}\frac{\left(P-P_{0}\right)}{4l}\right]}^{0.5}$ Palmqvist 0.25 < l/a < 2.5	$E=H_{0}\times \sqrt[{0.4}]{\frac{K_{I}\times a\times l^{0.5}}{0.0089}}$
0.491	4.38	1.49	11842.97	4.00	288
0.981	6.30	3.52	11447.81	3.17	278
1.962	9.22	7.35	10701.98	2.76	260
2.943	11.34	11.46	10612.68	2.62	258
4.903	14.66	16.51	10580.24	2.74	257
9.807	21.22	29.38	10100.5	2.78	246
19.610	30.05	46.81	10074.63	3.08	245
			**average value**	**3.02**	**261.8**
			**SD**	**0.44**	**14.80**

**Figure 18:**
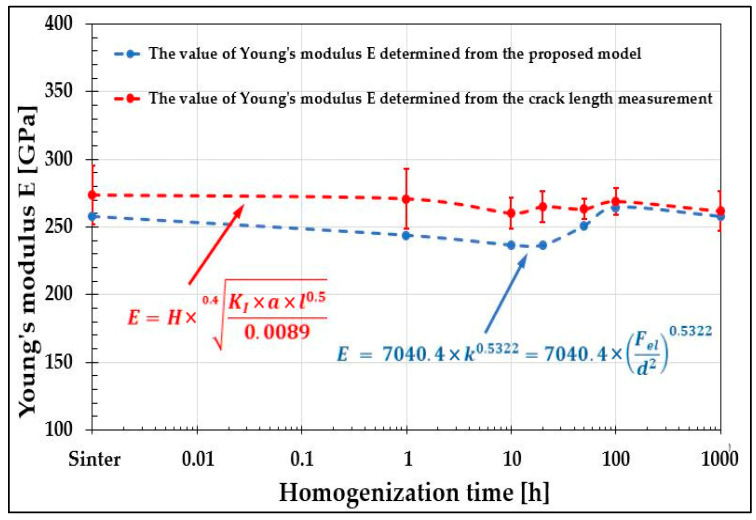
Comparison of the Young’s modulus E values determined for the TiCoCrFeMn alloy from microhardness measurements and from the analysis of models for determining fracture toughness using hardness measurements.

Comparison of the Young’s modulus values for the different states of the studied alloy (Figure 18) shows a very good correlation between the results obtained using both discussed methods. Small discrepancies are observed up to 100 h of homogenization, when phase transformations associated with fluctuations in chemical composition occur within the material. However, stabilization of the alloy structure, which takes place after 100 h of homogenization, results in practically identical values of Young’s modulus determined by the presented methods. This observation suggests that the proposed model for determining the material constant, specifically Young’s modulus, from microhardness measurements can be applied to small-sized samples that are not suitable for classical testing methods. Nevertheless, despite obtaining comparable results, to verify the accuracy of the analyses—particularly for the proposed model based on Meyer’s law and Kick’s law—it is necessary to determine the Young’s modulus of the produced alloys using recognized and validated experimental methods.

**Figure 19:**
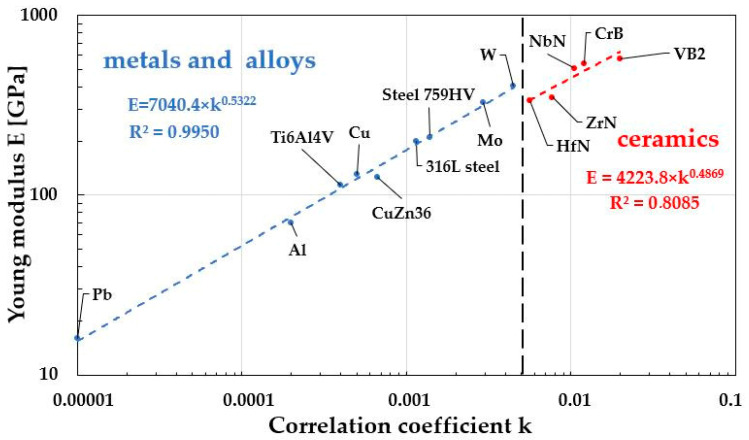
Differences in the characteristics of the dependence of the Young’s modulus E value as a function of the correlation coefficient “k” for metals and their alloys and ceramics.

The comparability of the results obtained for metals and their alloys, along with the high fit quality of the E–f(k) relationship (R^2^ = 0.995), also encouraged an attempt to determine this dependence for another material group—ceramics. However, the results obtained from literature data (Table 10) show a significantly lower fit, reaching only R^2^ = 0.8085 (Figure 19). It can also be observed that different material groups exhibit different functional dependencies characterizing E = f(k). This effect is undoubtedly related to the type of dominant atomic bonding in each material category. It should, of course, be noted that the presented relationships are incomplete. To refine them, a much larger number of measurements would be required, taking into account the influence of various material factors on both the Young’s modulus and the determined correlation coefficient “k,” such as the degree of deformation in metals and their alloys or the density and purity of ceramics.

**Table 10:** The correlation coefficients “k” and the corresponding values of Young’s modulus E for ceramics determined on the basis of literature data.

Ceramics			
	k	E [GPa]	References
**ZrN**	0.0077	351	[49]
**HfN**	0.0056	336	[49]
**CrB**	0.0120	542	[50]
**NbN**	0.0104	510	[51]
**VB_2_**	0.0199	577	[52]

## 4. Conclusions

1. Among the analyzed models for determining macroscopic hardness based on microhardness measurements, the model proposed by H. Li and R.C. Brandt, which accounts for the proportional increase in the elastic resistance of the sample during indenter penetration (referred to as the PSR model), exhibits the slightest deviation from the actual hardness values.
2. The literature-reported method for estimating the Young’s modulus from microhardness measurements using the Knoop method is associated with too large an error, making this model impractical for reliable use.
3. By applying Meyer’s law together with Kick’s similarity law, it is possible to determine from Vickers microhardness measurements the forces responsible for elastic deformation in the indentation area.
4. By determining, for the elastic strain influence zone (ISE), the correlation coefficient $k=\frac{F_{el}}{d^{2}}$, it is possible to establish the functional relationship:

$$E=7040.4\times k^{0.5322}=7040.4\times {\left(\frac{F_{el}}{d^{2}}\right)}^{0.5322}$$

For metals and their alloys, this relationship exhibits a high coefficient of determination R^2^ = 0.995, enabling the estimation of the Young’s modulus of newly designed engineering materials, such as high-entropy alloys, based on microhardness measurements.

## Figures and Tables

**Table 2:** The hardness values of the TiCoCrFeMn alloy determined for different models eliminating the effect of elastic deformation.

	Homogenization Time						
Model	Sinter	1 h	10 h	20 h	50 h	100 h	1000 h
Meyer’s law	1112	1102	1116	1106	1095	1140	1119
Hays-Kendall	1026	1051	1053	1059	1063	1082	1029
PSR	1002	1028	1043	1039	1047	1066	998
MPSR	934	1006	978	1026	1046	1011	956
Nix-Gao	1056	1013	1074	1067	1047	1082	1009

**Table 3:** The actual hardness values of the standards in the range of 30 kgf load, constituting a reference to the values determined from empirical models.

	Steel Hardness Standard of 228 HV0.1 ± 9	Steel Hardness Standard of 51.3 HRC	Steel Hardness Standard of 759 HV30 ± 16	Cemented Carbide
HV30 (measured)	217	526	759	1472
Standard deviation	±1	±1	±16	±18

**Table 4:** The values of the diagonal indentations obtained for the tested patterns as a function of the applied load.

Load F [gf]	Diagonal of the Hardness Indent of the Tested Material d [µm]			
	Steel Hardness Standard of 228 HV0.1 ± 9	Steel Hardness Standard of 51.3 HRC	Steel Hardness Standard of 759 HV30 ± 16	Cemented Carbide of 1472 HV30
10	8.42	-	3.85	-
25	13.42	8.85	6.10	-
50	19.64	12.45	8.62	7.17
100	28.25	17.92	12.58	10.34
200	40.13	25.90	18.37	15.00
300	49.49	31.84	22.47	18.54
500	63.60	41.13	29.34	24.25
1000	89.92	58.48	42.49	34.64

**Table 5:** The real hardness values of the standards determined from microhardness measurements based on the analyzed models compared to the hardness measured at a load of 30 kgf.

Empirical Model	Real Hardness HV			
	Steel Hardness Standard of 228 HV0.1 ± 9	Steel Hardness Standard of 51.3 HRC	Steel Hardness Standard of 759 HV30 ± 16	Cemented Carbide of 1472 HV30
Meyer’s law	244	570	880	1616
H-K model	228	540	828	1530
PSR model	225	530	815	1473
MPSR model	231	532	776	1460
HV30	216 ± 1	525 ± 1	743 ± 16	1454 ± 17

**Table 6:** Summary of the real hardness values determined from the analyzed models along with errors compared to the hardness of the standards measured at a load of 30 kgf.

		Steel Hardness Standard of 228 HV0.1	Steel Hardness Standard of 51.3 HRC	Steel Hardness Standard of 759 HV30	Cemented Carbide 1472 HV30	Average Value
Standard	HV30	217	526	759	1472	---
	SD	±1	±1	±16	±18	---
	Relative error	---	---	---	---	---
H-K Model	HV30	229	540	780	1510	---
	SD	±12	±14	±21	±38	---
	Relative error	5.46%	2.74%	2.80%	2.58%	**3.40%**
PSR Model	HV30	225	530	769	1465	---
	SD	±8	±4	±10	±7	---
	Relative error	3.63%	0.79%	1.28%	0.48%	**1.54%**
MPSR Model	HV30	231	533	776	1555	---
	SD	±14	±7	±17	±17	---
	Relative error	6.65%	1.27%	2.20%	1.15%	**2.28%**
N&G Model	HV30	222	532	780	1400	---
	SD	±5	±6	±21	±72	---
	Relative error	2.51%	1.11%	2.73%	4.89%	**2.81%**

**Table 7:** Data for determining the correlation coefficient “k” for the HV759HV steel hardness standard.

F [N]	d [µm]	d^2^ [µm^2^]	a	*n*	F_el_ [N]
0.09807	4.38	19.22	0.005794	1.926	0.011
0.2452	6.84	46.85	0.005794	1.926	0.035
0.4903	9.93	98.73	0.005794	1.926	0.088
0.9807	14.60	213.31	0.005794	1.926	0.219
1.961	20.91	437.40	0.005794	1.926	0.504
2.942	24.98	624.18	0.005794	1.926	0.757
4.903	32.93	1084.58	0.005794	1.926	1.415
9.807	47.31	2238.94	0.005794	1.926	3.184

**Table 8:** The determined correlation coefficients “k” and the corresponding values of Young’s modulus E for the analyzed engineering materials, as well as the values of Young’s modulus determined from the E = f(k) characteristic for the tested high-entropy TiCoCrFeMn alloy.

Metals and Their Alloys			Tested TiCoCrFeMn Alloy		
Material	k	E [GPa]	Homogenization Time	k	E [GPa]
**Al**	0.0002	70	**sinter**	0.0020	257.7
**Cu**	0.0005	130	**1 h**	0.0018	243.7
**Steel**	0.0014	210	**10 h**	0.0017	236.4
**Ti6Al4V**	0.0004	114	**20 h**	0.0017	236.4
**Mo**	0.00293	329	**50 h**	0.0019	250.8
**Pb**	0.00001	16	**100 h**	0.0021	264.5
**CuZn36**	0.00067	125	**1000 h**	0.0020	257.7
**316L**	0.00114	200	**E = 7040.4** **∗** **k^0.5322^**		
**W**	0.00350	405			

## Data Availability

The original contributions presented in this study are included in the article. Further inquiries can be directed to the corresponding author.
